# Chimeric engulfment receptors: A new cell therapy approach for SIV and HIV infection

**DOI:** 10.1016/j.omtm.2022.12.010

**Published:** 2023-01-03

**Authors:** Rimas J. Orentas

**Affiliations:** 1 Scientific Director, Caring Cross Professor, Department Pediatrics, University of Washington School of Medicine, Seattle, WA 98185, USA; 2 Investigator, Seattle Children’s Research Institute, Seattle, WA 98101, USA

In this issue, authors Daniel Corey, Francoise Haeseleer, Joe Hou, and Lawrence Corey from CERo Therapeutics and the Fred Hutchinson Cancer Center describe a new approach to creating chimeric antigen receptors (CARs) in “Chimeric engulfment receptors (CERS): a new cell therapy approach for SIV- and HIV-infection”.^1^ Effective disease control with engineered T cells against targets other than hematologic malignancies, whether the application is for solid tumors or viral-specific CARs, remains elusive. Viral-specific T cells for cytomegalovirus (CMV) and Ebola virus (EBV) have been shown to be effective in some settings. Activity against HIV, whether mediated by a T cell receptor (TCR)- or CAR-based approach has proven to be much more difficult. In an entirely orthogonal approach, Corey et al. targeted a characteristic of an infected cell population as opposed to an antigen encoded by the virus itself or an overexpressed tumor-associated protein.^1^ The expression of phosphatidyl serine (PS) on the surface of effete red cells, apoptotic cells, and tumor cells is well established. Corey et al. observed that PS is also overexpressed on cells infected by lentiviruses early post-infection. The cell surface glycoprotein TIM-4 specifically binds PS. Expression of TIM-4 by macrophages facilitates the clearance of apoptotic cells and the engulfment of effete red cells, removing them from circulation. Corey et al. linked the extracellular aspect of TIM-4 that binds to PS with intracellular signaling domains normally associated with CAR-T activity, thus creating chimeric “engulfment” receptors (CERs). CERs were demonstrated to mediate enhanced killing of simian immunodeficiency virus (SIV)-infected CD4+ T cells, demonstrating that an innate immune receptor system can be redirected to clear virus-infected cells.

Expression of PS on the cell surface can be induced by cells whose biology has been altered by viral or parasitic infection. HIV specifically triggers the expression of PS early in infection not only through a cell death pathway but by a much earlier event whereby scramblase enzymes (which redistribute phospholipids on the membrane) are activated in order to promote membrane fusion events required for infection and viral budding.^2^

Building on these observations, Corey et al. designed CERs to take advantage of the induced exposure of PS, leveraging the ability of TIM-4 to bind PS and thereby transmit a CAR-like transmembrane signaling cascade.^3^^,^^4^ Another novel aspect of developing the reported CER constructs was the screening assays carried out to evaluate both standard T cell activation domains often included in CARs, such as CD3-zeta or CD28, as well as signaling receptors from innate immune receptors involved in pathogen recognition. These included the signaling domains from Toll-like receptors (TLRs), TRAF6, DAP10, and DAP12. The greatest anti-SIV effects were seen with CER constructs expressing either TLR8-CD3zeta or TRAF6 intracellular signaling domains. The TLR8 signaling pathway includes MyD88 and TRAF6, both resulting in nuclear factor κB (NF-κB) expression and cellular activation.

While the CER approach is novel, the mechanism of cell killing has yet to be established. CERs are active in CD4 T cells but not CD8 cells, which has not been reported in either CAR or recombinant TCR approaches. Thus, the activated T cells may be activated to kill their PS-expressing targets by expression of NK-like receptors, i.e., CTAK activity, by nibbling of target cell membranes or by a yet-to-be-described mechanism of membrane alteration.^5^^,^^6^ Linking of TLR8-CD3zeta or TRAF6 signaling to a specific killing mechanism would allow for a broader exploration of the reported findings.

The significance of this new work lies in the demonstrated ability to engineer cells of the adaptive immune system to recognize cellular characteristics normally associated with innate immune cell activity. Corey et al. used PS as a signal to control retroviral infection by an engineered T cell. Similar receptors, if expressed by T regulatory cells, could be used to modify tissue inflammation. Future work could use a cascade of these signals to gate effector or regulatory functions that react to a more broadly expressed target cell characteristics that reflect cellular physiology as opposed to tradition microbial or cancer-associated antigens. Immediate next steps are to test the SIV model presented *in vivo* to determine if CER-T cells can control SIV infection in an animal model.
